# Grazing practice affects the growth performance, meat quality and nutritional composition, and fecal microbiota of fattening yaks

**DOI:** 10.5713/ab.25.0052

**Published:** 2025-06-04

**Authors:** Kewei Hu, Qi Wu, Tianxiang Chen, Jiakun Wang, Zhixiang Zhu, Yulei Shen, Chong Wang, Xiaoshi Wei

**Affiliations:** 1 College of Animal Science and Technology & College of Veterinary Medicine, Zhejiang A&F University, Key Laboratory of Applied Technology on Green-Eco-Healthy Animal Husbandry of Zhejiang Province, Hangzhou, China; 2 Animal Husbandry and Veterinary Service Center, Ruoergai, China; 3 College of Animal Science, Zheijiang University, Hangzhou, China; 4 Ruoergai Grassland Dawn Feeds Co., Ltd., Ruoergai Grassland Dawn Modern Livestock Co., Ltd., Ruoergai, China

**Keywords:** Fecal Microbiota, Grazing Practice, Meat Nutritional Composition, Shelf Life, Yak

## Abstract

**Objective:**

Livestock grazing is the primary practice in alpine meadows, which is closely related to animal performance and ecosystem functions. This study aimed to evaluate the effects of grazing practice on the growth performance, meat nutritional composition and shelf life, and fecal nutrient and microbiota of yaks.

**Methods:**

Twenty-four male yaks (217.62±5.74 kg) were randomly divided into 2 groups for a 60 d fattening experiment: grazing (G) group and grazing and supplementary feeding (GS) group. The yaks in the G group were grazed only on pastures, without any supplements. The yaks in the GS group were not only grazed on natural pastures, also supplemented with the concentrate mix based on the body weight after grazing.

**Results:**

Supplementary feeding concentrate mix after grazing significantly increased the body weight (p<0.01) and average daily gain (p<0.01) of yaks. The results indicated that supplementary feeding reduced meat shear force (p = 0.04), increased the a* value (p<0.01), the b* value (p = 0.04) and the ether extract content (p = 0.03), and extended the shelf life by 3.4 h. The total amino acid content increased (p<0.01) and promoted the deposition of monounsaturated fatty acids (p<0.01) and polyunsaturated fatty acids (p<0.01). The output concentration of nitrogen in feces was increased (p = 0.04), and the 16S rRNA sequencing results showed that grazing with supplementary feeding significantly increased the relative abundance of key genera, including *Alistipes*, UCG-009, *Tuzzerella*, Family_XIII_UCG-001, and *Erysipelatoclostridium*, which are associated with nutrient absorption, fiber degradation, and metabolism.

**Conclusion:**

Post-grazing concentrate mix supplementation improved yak growth, meat quality, and shelf life, likely via enhanced amino acid and fatty acid deposition, nitrogen retention, and gut microbial shifts, which may ffers new insights into nutrient metabolism and feeding strategies for high-altitude livestock.

## INTRODUCTION

The yak (*Bos grunniens*) is an indigenous herbivore raised in the Asian highlands between 3,000 and 5,000 meters above sea level [1]. Approximately 90% of the world’s yaks are found in China, where they provide over 90% of the milk and dairy products and 50% of the meat for local people, along with leather, transportation and dung fuel [2]. Most yaks are still kept under traditional management, grazing on highland pastures throughout the year. However, the plateau’s cold climate, which lasts about seven to eight months per year, with average temperatures ranging from −15°C to −5°C, severely limits forage availability, causing yaks to lose 25% of their original body weight (BW) [3]. Moreover, overgrazing contributes to severe degradation and desertification of large portions of alpine meadows.

Compared to commercial beef from lowland cattle, yak meat is of higher quality because it is low in fat, high in protein and rich in minerals, essential amino acids (EAA) and polyunsaturated fatty acids (PUFA) [4]. With the improvement of living standards, consumers are increasingly prioritizing the quality and health attributes of food when choosing [5], also the appearance, color, and sensory characteristics. Despite the high consumer demand for yak meat, the industry faces production inefficiencies, leading to tight supply and demand. Supplementation with high-energy diets after grazing has been shown to improve growth performance, rumen microbial protein synthesis, antioxidant capacity and immunity in growing yaks [6], and supplementing mixed diets in winter effectively improved the growth and slaughter performance of Tibetan sheep, also the meat quality [7]. Whether summer grazing practices affect meat quality and nutritional composition in yaks remains to be elucidated.

Moreover, as the key biological component of the plateau ecosystem, Yak waste significantly impacts the local nitrogen (N) cycle and the broader environment. They incorporate N from plants into their bodies, and excrete nitrogenous compounds that enrich the soil and promote plant growth. This process is essential for maintaining soil fertility and the overall health of the grassland ecosystem. In addition, the grazing practices affect soil microbial networks [8].

We hypothesized that changing grazing practices could affect growth performance and meat quality, and be potential to the ecosystem. The aims of this study were to evaluate the effects of grazing parctice on the growth performance, meat quality and nutritional composition of yaks, also the changes in fecal volatile constant element and bacterial composition. These results could help us understand the role of grazing practice in yaks, while also providing an experimental basis for the grazing management and scientific feeding of yaks.

## MATERIALS AND METHODS

### Animals and experimental design

This study was conducted at the Ruoergai Yak Breeding Ranch No. 1 (33.58°N, 102.95°E) in Sichuan, China. The average temperature during the experimental period was 15.8°C, the average rainfall was 110–120 mm, and the average elevation was 3,406 m.

A total of twenty-four healthy male yaks (with the same genetic background; age = 5±0.5 years old, BW = 217.62±5.74 kg) were selected, dewormed prior to the experiment, and randomly assigned to two groups. The yaks were randomly assigned into two experimental groups, each containing 12 individuals as biological replicates. The sample size was chosen based on the expected effect size, ensuring sufficient statistical power to detect significant differences in key variables such as BW gain, meat quality, and nutritional composition. The yaks were randomly assigned into two experimental groups, with each group containing 12 individuals as biological replicates. The grazing group (G): yaks were grazed on natural pasture without supplements. The grazing and supplementary feeding group (GS): yaks were grazed on natural pastures and fed supplementary concentrate mix after grazing. The amount of concentrate mix was adjusted according to BW, with daily intake monitored by recording feed offered and refusals. Adjustments were made weekly to ensure nutritional requirements were met.

The supplementary concentrate mix was formulated on a dry matter (DM) basis and primarily consisted of 63.5% corn grain, 11.5% rapeseed meal, 10% wheat bran, 8% cottonseed meal, 3.5% soybean meal, 1.5% calcium hydrogen phosphate, 1% sodium chloride, and 1% mineral–vitamin premix. Its nutrient composition included 91% DM, 17.1% crude protein (CP), 2.7% ether extract (EE), 29.4% neutral detergent fiber (NDF), 16.7% acid detergent fiber (ADF), 0.7% calcium (Ca), and 0.4% phosphorus (P). As the experiment was conducted in July, the forage quality was at its peak. The nutrient composition of the alpine pasture included 91.9% DM, 9.7% CP, 2.2% EE, 54.4% NDF, 34.5% ADF, 1.6% Ca, and 0.5% P. All yaks had free access to grazing and water throughout the study period.

### Sample collection

#### Feed

At the beginning and end of the experiment, 50 g of experimental feed was homogenized, dried at 60°C for 48 h, pulverized using a high-speed grinder, and passed through a 2 mm sieve. The processed sample was then immediately vacuum-sealed and frozen at −20°C for subsequent chemical analysis.

#### Body weight

The experimental yaks were weighed at the start, mid-point (30 d), and end (60 d) of the experiment to calculate the average daily gain (ADG) for each individual.

#### Meat

At the end of the experiment, all yaks were humanely slaughtered by certified technicians in accordance with the relevant regulations outlined in the Animal Epidemic Prevention Law and the Food Safety Law of the People’s Republic of China, at a licensed commercial slaughterhouse certified by local authorities, ensuring compliance with local animal welfare standards. The yaks were fasted for 13 h prior to slaughter to ensure a standardized pre-slaughter condition and minimize the influence of digestive contents on meat quality. Longissimus dorsi (LD) samples were obtained from the 12 to 13th intercostal space on the left side of the carcass immediately post-slaughter. LD sample was divided into three 3-cm-thick sub-samples. One sub-sample was stored in a plastic container wrapped with polyethylene film for the evaluation of meat color, pH, water-holding capacity, and shear force, while another sub-sample was designated for shelf-life assessment. The storage conditions were closely monitored using calibrated temperature sensors to ensure consistent environmental control. The third sub-sample was vacuum-packed and frozen at −20°C for long-term biochemical analysis. Freezing at this temperature effectively prevents enzymatic and microbial activity, preserving the integrity of the sample for subsequent tests.

#### Faece

50 g fecal samples were collected on the day before slaughter. Samples were immediately transferred to a portable cooler with ice packs and transported to the laboratory within 2 h. They were then stored at −20°C for subsequent microbiological and chemical analyses.

### Determination of nutrients in feed, meat and faeces

Meat and fecal samples were dried at 65°C for 72 h using a forced-air drying oven and ground to pass through a 1 mm sieve using a Wiley mill. DM was determined by drying feed samples at 105°C for 24 h in a drying oven. Organic matter content was measured after combustion at 550°C for 8 h in a muffle furnace, ensuring complete oxidation. Total N content was analyzed using the micro-Kjeldahl method, involving digestion with concentrated sulfuric acid and copper sulfate as a catalyst, followed by steam distillation and titration with 0.01 N HCl. CP was calculated as N×6.25, with calibration performed using a N standard (ammonium sulfate). NDF and ADF were determined using the AOAC method [9]. Samples were sequentially treated with neutral detergent solution and acid detergent solution, followed by ashing at 550°C to correct for ash content. EE content was measured using a Soxhlet extraction system with petroleum ether (boiling range: 40°C–60°C) at 90°C for 1 h. All analyses were conducted in triplicate to ensure accuracy and reproducibility, with standard materials and procedural blanks included for quality control.

### Meat quality determination

For pH measurement, each sample’s pH was determined at 45 min and 24 h post-slaughter at 4°C using a portable pH meter with a glass electrode (model XYZ). The pH meter was calibrated with standard buffer solutions (pH 7.0) prior to each use. Meat color was assessed at the same time using a CR-400 colorimeter, recording L* (lightness), a* (redness), and b* (yellowness). Samples were exposed to air for 30 min at 4°C before recording L*, a*, and b* values at three locations per sample. Cooking loss was measured as the percentage weight difference before and after cooking samples in an 80°C water bath for 30 min, following Chinese National Standard NY/T 821-2019. Drip loss was calculated from the weight difference of LD muscle samples stored at 4°C on days 1 and 6, with samples suspended in airtight containers to prevent reabsorption. Shear force was determined using a Warner-Bratzler shear force device at a crosshead speed of 2 mm/s on 1 cm×1 cm×3 cm strips, aligned parallel to the muscle fibers. Each measurement was performed in triplicate, with mean values used for analysis.

### Shelf-life determination

Shelf life of meat was determined by measuring total volatile basic nitrogen (TVB-N) content using an automatic Kjeldahl nitrogen analyzer (model K9840; Hanon). The analyzer was calibrated with ammonium sulfate standards prior to each batch. At each time point (24, 48, 72, 96, and 120 h), independent samples were analyzed in triplicate to avoid cross-contamination. Samples were homogenized, and 10 g aliquots were treated with magnesium oxide and distilled water before distillation. Volatile nitrogen in the distillate was titrated with 0.01 N sulfuric acid, and results were expressed as mg TVB-N per 100 g of meat. Samples were stored in a ventilated cold room maintained at 4°C with 85% relative humidity to simulate typical refrigerated conditions. The specific detection procedure followed Bai et al [10], which involved standard Kjeldahl nitrogen distillation and titration techniques. All measurements were performed in triplicate, with mean values and standard errors reported.

### Faeces volatile fatty acids in the faeces

The concentrations of fecal volatile fatty acids (VFA) were determined according to Liu et al [11]. The analysis was performed using gas chromatography equipped with a capillary column (AT-FFAP; 30 m×0.32 mm×0.5 μm).

### Amino acids in the longissimus dorsi muscle

Based on the method described by Vopálenský et al [12], the amino acid (AA) content was determined using an automatic AA analyzer (model L-8900; Hitachi). This method relied on the colorimetric reaction between AAs and the oxidizing agent ninhydrin. Amino acids in the DM of the samples were quantified after acidic hydrolysis in 6 N HCl for 24 h under controlled conditions.

### Fatty acids in the longissimus dorsi muscle

Determination of the absolute content of fatty acids (FAs) in yak LD: (1) Lipids were extracted from yak LD using Soxhlet extraction with chloroform–methanol (2:1, v/v) three times and dried under nitrogen. (2) Extracted lipids were hydrolyzed to free FAs using a sodium hydroxide–methanol solution. (3) FAs were esterified with a boron fluoride–methanol solution to form fatty acid methyl esters (FAME). FAMEs were extracted with n–heptane, dried under nitrogen, redissolved, and analyzed using gas chromatography. Analytes were identified by comparing retention times with standards. Absolute FA content was calculated from FAMEs, and relative content was determined as:


(1) (Absolute content of a specific FA)/(Absolute content of total FAs)×100.

### Fecal microorganism DNA extraction and polymerase chain reaction amplification

Microbial DNA was extracted from feces using the E.Z.N.A. Soil DNA Kit (Omega Bio-tek). DNA quality was verified via agarose gel electrophoresis and quantified using NanoDrop 2000. The V3-V4 region of the 16S rRNA gene was amplified with barcoded primers (338F and 806R). Polymerase chain reaction conditions included 27 cycles of denaturation, annealing, and extension, with products purified using a gel extraction kit and quantified with Qubit 4.0. Sequencing data were processed via fastp and FLASH for QC, merging, and filtering. DADA2 in QIIME2 was used for denoising, and taxonomy was assigned using the SILVA database (v138). PICRUSt2 was employed to predict functional pathways based on 16S rRNA data.

### Statistical analysis

The experimental data in this study were statistically analyzed using SPSS 26.0 (IBM). For comparisons between groups, an independent-samples t-test was used. Growth performance data with repeated measurements were analyzed using a linear mixed-effects model, in which group and time were treated as fixed effects, and individual yak was treated as a random effect. Time was also specified as the repeated measure. The individual yak was considered the experimental unit. All data are presented as means±standard error of the mean (SEM). Statistical significance was set at p<0.05. In addition, effect sizes were calculated using Cohen’s d, defined as the difference between the two group means divided by the pooled standard deviation.

## RESULTS

### Growth performance

The growth performance recorded during the experiment is summarized in Table 1. Interval BW (measured on day 30 of the 60-day trial), final BW (day 60), and ADG were significantly higher (p<0.001) in yaks from the GS group compared to those in the G group. The corresponding effect sizes (Cohen’s d) for interval BW, final BW, and ADG were 2.742, 6.201, and 7.939, respectively. Additionally, the mixed model analysis indicated a significant interaction effect of Group×Time (p<0.001) on these variables.

### Meat quality, chemical composition, and shelf life

The results of meat quality analysis of yaks in the G and GS groups are presented in Table 2. The yaks in the GS group had significantly higher a* (p = 0.008; Cohen’s d = 0.947) and b* (p = 0.039; Cohen’s d = 0.686) values, and significantly lower shear force (p = 0.047; Cohen’s d = 0.666) compared to those in the G group. No significant differences were found in final pH or L* values between the two groups. Chemical composition analysis revealed that the EE content was significantly higher (p = 0.030; Cohen’s d = 0.790) in yaks from the GS group compared to those in the G group. The moisture, CP, and ash contents did not show significant differences between the two groups.

**Figure 1:**
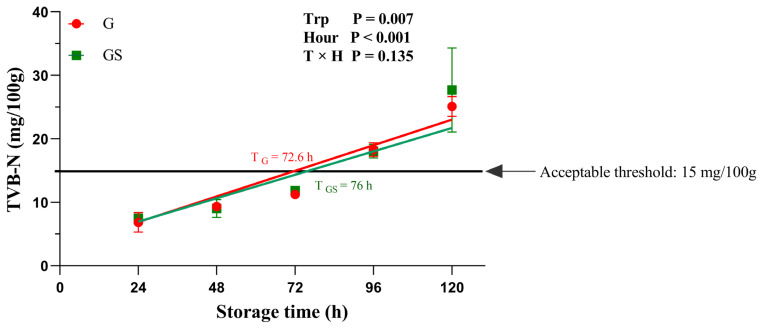
Effect of different grazing practice on the shelf life of yak meat. The red line and circles represent yaks in the G group, while the green line and squares represent yaks in the GS group. Values of TVB-N are presented as means±SEM, derived from three independent experiments. The horizontal line represents the acceptable threshold for fresh meat TVB-N content (15 mg/100 g) according to China's national standard GB 2707-2016. p-values for group effect (Trp), time effect (Hour), and their interaction (Trp×Hour) are shown in the graph and were analyzed using a linear mixed model. G, grazing group; GS, grazing and supplementary feeding group; TVB-N, total volatile base nitrogen; SEM, standard error of the mean.

Figure 1 illustrates the changes in meat shelf life based on TVB-N content. Both groups showed increased TVB-N over time (p<0.001), with GS yaks exhibiting a significantly lower TVB-N levels than G yaks (p = 0.007). The shelf life threshold (15 mg/100 g) was reached at 72.6 h and 76 h for G and GS, respectively. No significant interaction between treatment and storage time was observed.

### Content of amino acids

The results of the absolute AA content analysis in LD are presented in Table 3. A total of 17 AAs were detected in the LD of both the yaks in G and GS group. The levels of Thr, Glu, GIY, Cys, Iso, and total AAs were significantly higher in the yaks in GS group compared to the yaks in G group (p<0.05).

### Content of fatty acid

The results of absolute FAs content analysis in LD are presented in Table 4. A total of 31 FAs were detected in the LD of both the yaks in G and GS yaks, comprising 15 saturated fatty acids (SFA), 10 monounsaturated fatty acids (MUFA), and 6 PUFA. The absolute content of C16:0, C17:0, C20:0, C26:0, C10:1, C14:1, C15:1, C17:1, C18:1, C19:1, C20:1, C22:1, C18:2, C20:4, C22:6, total PUFA and total unsaturated fatty acids (UFA) of the yaks in GS were higher than those the yaks in G group (p<0.05), whereas the absolute contents of C6:0, C8:0, C12:0, C13:0, C14:0, C15:0, C18:0, C19:0, C24:0, and total MUFA were lower than those values in the LD of yaks in G group (p<0.05).

### Chemical analysis and volatile fatty acids of feces

The constant elements and VFA of yak feces are presented in Table 5. The results showed that supplemental concentrate mix significantly increased the levels of Ca (p = 0.013; Cohen’s d = 0.867) and N (p = 0.045; Cohen’s d = 0.178) in yak feces. There were no significant changes in fecal levels of P. Regarding VFA, no significant differences were observed in the individual VFA concentrations or total VFA levels between the yaks G and GS groups. The acetate-to-propionate ratio (A: P) also showed no significant change.

### Fecal alpha diversity

The alpha diversity indices (ACE, Chao, Shannon, Simpson, and Sobs) in yak feces under different grazing practices are presented in Table 6. The results showed no significant differences in any of the diversity indices between the Yaks in G and GS groups.

### Fecal bacteria structure

**Figure 2:**
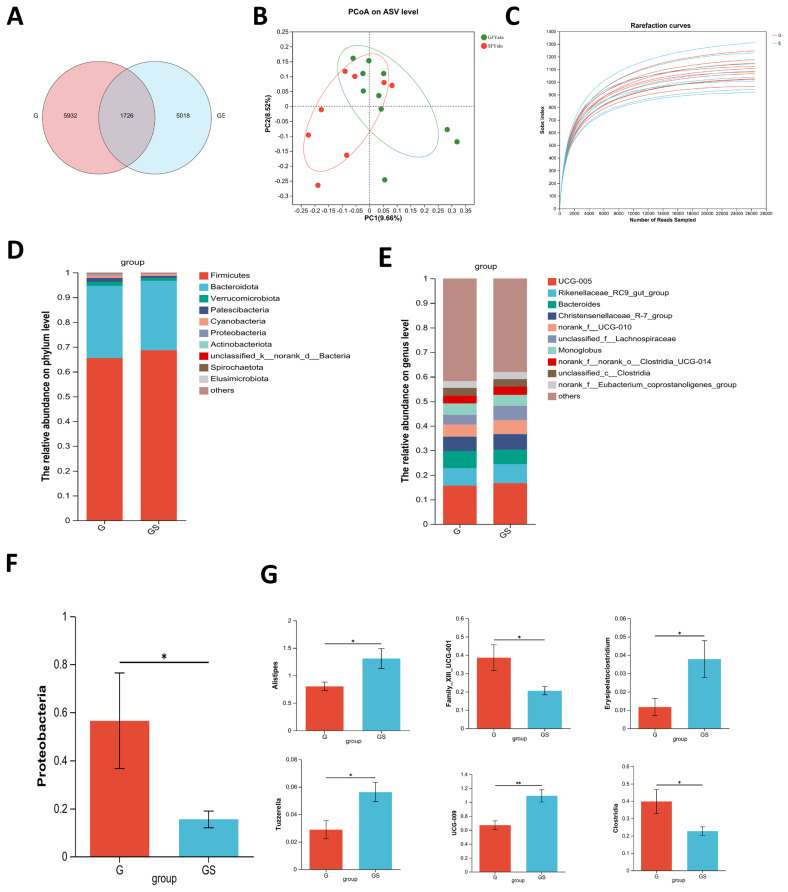
Comparison of fecal microbiota composition and diversity between the yaks in G group and yaks in GS group. (A) Venn diagrams of the fecal bacterial community between the yaks in G and GS group. (B) Principal coordinate analysis (PCoA) with fecal bacterial community between the yaks in G and GS group. (C) Rarefaction curves for the G and GS groups showing the relationship between sequencing depth (number of reads) and observed species richness (Sobs index). (D) Fecal bacterial composition at the phylum level (the relative abundance>0.5%) in the yaks from G and GS groups. (E) Fecal bacterial composition at the genus level (top 10) in the yaks from G and GS groups. (F) Relative abundance of significantly different bacteria at the phylum level between G and GS groups. (G) Relative abundance of significantly different bacteria at the genus level between G and GS groups. A single asterisk (*) indicates a significant difference at p<0.05, while double asterisks (**) indicate a significant difference at p<0.01. G, grazing group; GS, grazing and supplementary feeding group.

A Venn diagram showed 1,726 common OTUs in fecal samples of both groups, with 5,932 unique OTUs identified in the yaks in the G group and 5,018 in the GS group, respectively (Figure 2A). PCoA plots of microbial compositions in the yaks in G group and yaks in GS groups, generated using ANOSIM analysis based on Bray–Curtis distance, revealed significant differences in fecal bacterial structure between the two groups (p = 0.02, Figure 2B). Rarefaction curves for the G and GS groups were generated to evaluate the sequencing depth and observed species richness (Sobs index). Both groups exhibited a plateau as sequencing depth increased, indicating that the sequencing depth was sufficient to capture the microbial diversity present in the fecal samples of both groups (Figure 2C). At the phylum level, *Firmicutes* and *Bacteroidetes* were predominant in both groups (Figure 2D), with the yaks in G group having a significantly higher relative abundance of *Proteobacteria* compared to the yaks in GS group (p*<*0.05, Figure 2F). Fecal bacterial composition at the genus level, showing the top 10 genera in both groups (Figure 2E). At the genus level, significant differences were observed in *Alistipes* (p = 0.03), *UCG-009* (p*<*0.01), norank_c__*Clostridia* (p = 0.04), Family_XIII_UCG-001 (p = 0.03), *Tuzzerella* (p = 0.01), and *Erysipelatoclostridium* (p = 0.04, Figure 2G).

### Correlation analysis among different fecal bacteria and volatile fatty acids

**Figure 3:**
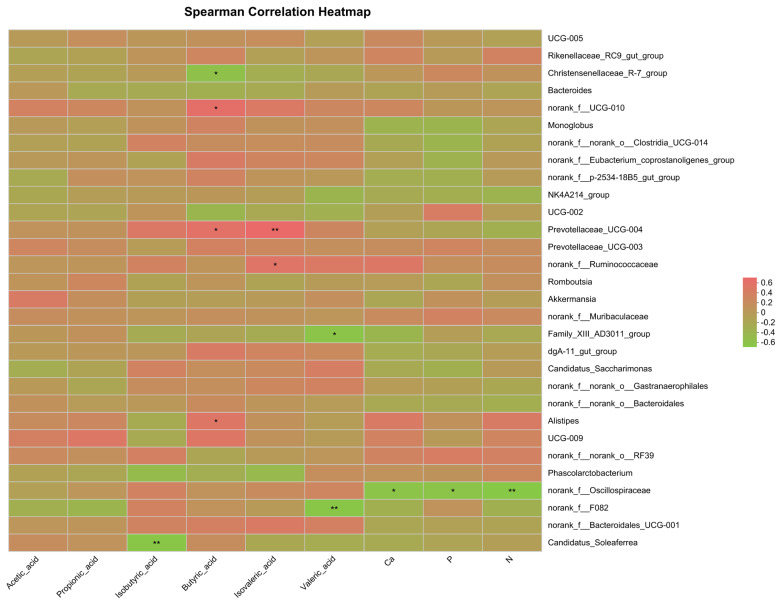
Heatmap diagram of correlations between fecal bacteria at genus level and volatile fatty acids. Red was negatively correlated and green was positively correlated. Correlation significance p-value was indicated by “*”. * 0.01<p≤0.05; ** 0.001<p≤ 0.01.

Figure 3 illustrates the correlations between the relative abundance of the top 30 fecal bacterial genera, VFA, and minerals such as Ca and P. Spearman’s correlation analysis showed that norank_f__*Oscillospiraceae* showed a significant negative correlation with Ca (R = −0.633, p = 0.011, R^2^ = 0.400), P (R = −0.622, p = 0.013, R^2^ = 0.387), and N (R = −0.692, p = 0.004, R^2^ = 0.479). Prevotellaceae_UCG-004 was positively correlated with butyric acid (R = 0.579, p = 0.024, R^2^ = 0.335) and isovaleric acid (R = 0.688, p = 0.005, R^2^ = 0.473). Christensenellaceae_R-7_group was negatively correlated with butyric acid (R = −0.582, p = 0.022, R^2^ = 0.339). The relative abundance of *Alistipes*, which increased in the GS group, showed a weak positive correlation with butyric acid (R = 0.516, p = 0.048, R^2^ = 0.266). In contrast, Candidatus_*Soleaferrea* exhibited a significant negative correlation with isobutyric acid (R = −0.662, p = 0.007, R^2^ = 0.438).

## DISCUSSION

One of the objectives of this study was to examine the effects of different farming modes on growth performance, meat quality, and fecal status of yaks. Growth performance is an important indicator reflecting the level of livestock production [13]. Chikwanha et al [14] reported that livestock growth and slaughter performance improve with increased dietary nutrient levels. Wang et al [7] found that higher dietary concentrate-to-coarse ratios improved growth and slaughter performance in Tibetan sheep. In this study, the nutritional composition (e.g., CP and EE) of supplementary feed was significantly better than that of natural forage, and the nutrient intake of the yaks in GS group was significantly higher than that of yaks in G group. This suggests that when the daily nutrient intake of livestock is sufficient, they can better convert the excess nutrients into growth and slaughter performance beyond the basic metabolism needed to maintain daily activities.

Tenderness, a key beef quality characteristic, is measured by shear force, with lower values indicating better tenderness [15]. Nuernberg et al [16] showed that animals fed a concentrate mix have lower shear force values compared to those fed grass-based diets. Keller et al [17] also observed that concentrate-fed cattle exhibited higher intramuscular fat (IMF) levels, which could enhance meat tenderness by improving fat deposition in muscle tissue, making the meat softer and easier to chew. This finding is consistent with our study, where yaks supplemented with concentrate mix exhibited significantly lower shear force compared to the yaks in G group. This suggests that concentrate mix supplementation positively affects meat tenderness. The improvement in shear force may be attributed to the enhanced deposition of IMF, as the protein, fat, and other energy-rich components of the concentrate mix may promote the production of glycerol and FAs, leading to greater IMF accumulation in muscle tissue and a corresponding decrease in shear force.

Meat color is a crucial indicator of meat’s appearance and product value. Lipid oxidation, closely associated with meat color, generates free radicals that increase ferrimyoglobin formation, thereby reducing color stability [18]. In this study, concentrate mix supplementation elevated a* and b* values without altering the L* value. This could be attributed to the enhancement of muscle antioxidant function by vitamin E and flavonoids in the feed, which increase antioxidant activity. Vitamin E mitigates lipoperoxide formation and inhibits oxidation of PUFAS. Carotenoids, which deposit in fat tissue, enhance the yellow hue of meat [19].

TVB-N is a key indicator of meat freshness, with higher levels indicating shorter shelf life due to bacterial proliferation [20]. In this study, yaks in the GS group had a longer shelf life than the yaks in G group by approximately 3.4 h. This aligns with Wood et al [21], who found that feed composition influences meat quality and shelf life. The addition of concentrate mix likely altered the FAs composition, increasing UFA and reducing saturated ones, thus improving oxidative stability. In addition, concentrates rich in antioxidants, such as vitamin E, play a crucial role in slowing the oxidation rate of beef, as antioxidants are known to inhibit lipid peroxidation [22]. This antioxidant effect may be a key factor in the observed extension of shelf life.

The type and content of free amino acids (FAA) in muscle tissue profoundly influence its nutritional value and flavor. Higher FAA content enriches the nutritional value of food. For instance, Glutamate (Glu) and Cysteine (Cys) are essential for metabolic processes [23,24]. This study found increased concentrations of EAA and functional AAs (Asp, Glu) of the yaks in GS group, suggesting that supplemental concentrate mix enhances yak meat’s nutritional quality. Additionally, AAs like Asp and Glu, which participate in the Maillard reaction, are crucial for meat flavor [25]. Glycine enhances meat-like taste by reacting with reducing sugars [26]. Consistent with the findings of Zhang et al [27], our study also demonstrated that the supplementation of concentrate mix significantly increased the concentration of AAs in yak meat. However, unlike their findings on AA concentrations, the difference in our results lie in the higher concentration of Asp and Ile, which may be attributed to differences in the composition of the concentrate mix. The higher levels of Asp could be attributed to the increased N availability provided by the concentrate mix in the GS group, which likely supported AA synthesis. Similarly, the increased Iso concentration may reflect enhanced protein metabolism associated with the higher energy intake from supplementary feeding, improving muscle protein synthesis and AA profiles in the yak meat. The concentrate mix improved flavor-related AAs in yak muscle, enhancing marbling and taste. This suggests that concentrate mix can enhance FAA enrichment and meat quality by affecting nitrogenous compound utilization and protein conversion.

FAs in muscle significantly influence meat flavor and human health. Beef FAs are mainly classified into SFA, MUFA, and PUFA [28]. Key SFAs in ruminant meat include myristic (C14:0), palmitic (C16:0), and stearic (C18:0) acids [29]. Consumption of long-chain SFAs (C12:0 to C18:0) is linked to higher coronary heart disease risk. Lauric acid (C12:0) raises LDL cholesterol levels, while myristic (C14:0) and palmitic (C16:0) acids similarly affect cholesterol concentrations [30]. Stearic acid (C18:0) is metabolized into oleic acid (C18:1 n-9), which does not adversely impact cardiovascular risk [31]. Grass-fed beef typically has higher stearic acid levels but overall more favorable SFA composition [32,33]. Our study found that yaks supplemented with concentrate mix had significantly lower total SFAs compared to grass-fed yaks. MUFA has cardioprotective effects and regulates blood lipids, while PUFA reduces blood lipids, inhibits platelet aggregation, and supports growth [25]. This study showed that concentrate mix increased MUFA and PUFA levels in yak LD muscle. Grain feeding lowers rumen pH, reducing UFAs through biohydrogenation, thus increasing MUFA production [34]. Grass-fed beef’s lower fat content explains its lower PUFA levels [33]. Antioxidant components in the concentrate mix, such as flavonoids and polysaccharides, may protect UFAs and enhance FA profiles in yak meat. Carbonero et al [24] suggested that concentrate mix can improve the nutritional quality and flavor of yak meat by enhancing the FA profile, contributing to better dietary health benefits.

The interaction between pastures and grazing animals is critical for the health and sustainability of alpine grassland ecosystems. Soil, acting as the substrate for plant growth, stores essential N and P nutrients, influencing soil properties and regulating the ecological structure, function, and productivity of grasslands [35]. Nitrogen, a limiting factor for plant and microbial growth, primarily originates from soil N supply and atmospheric deposition in the Qinghai-Tibet Plateau, with fecal reflux as a significant source [36]. Yaks play a crucial role in the plateau’s N cycle. N in their feces can leach into the soil, influencing soil N processes, vegetation growth, and soil quality. Yang et al [37] found that supplementing with oat hay increased litter production and fecal reflux, enhancing soil N input. Our study corroborates this, showing that concentrate mix supplementation significantly elevated N content in feces. This suggests that concentrate mix enhances N retention and processing within the digestive system, reducing N excretion per unit of feed intake and increasing N concentration in manure. Consequently, more N is made available to the soil when manure is applied, enhancing N cycling efficiency and potentially increasing plant N availability.

However, P levels in feces did not show significant changes, which may be due to the more efficient recycling of P in ruminants. Unlike N, P is less likely to be excreted in excess due to its tight regulation in the digestive system and its limited bioavailability in plant-based feeds [38,39]. Furthermore, while N is highly influenced by dietary supplementation, P levels tend to be more stable, as it is often recycled through the rumen and retained by the animal [40]. This suggests that concentrate mix may not significantly alter P excretion, in contrast to its effect on N cycling. Additionally, we observed that Ca levels in feces increased with concentrate mix supplementation, potentially due to the higher mineral content in the concentrate. This suggests that dietary supplementation not only affects N cycling but also has implications for other mineral balances. Further studies are needed to explore the dynamics of P and Ca, as well as their interaction with different feed types in ruminant systems.

The microbial composition and diversity of bacteria in yak feces may be related to pasture environment, pasture quality, and species. Henderson et al [41] concluded that the dominance of *Firmicutes* and *Bacteroidetes* may result from changes in diet and climate. In this study, *Firmicutes* and *Bacteroidetes* were the dominant bacterial groups in both yak groups, indicating their significant roles in the yak posterior gut. *Proteobacteria* serve as a microbial signature of ecological dysbiosis in the gut microbiota. Shin et al [42] reported that a high relative abundance of *Proteobacteria* might cause gut inflammation. In our study, the relative abundance of *Proteobacteria* was higher in yaks in GS group than in the G group. This increase may be due to the concentrate mix altering nutrient availability and pH, favoring *Proteobacteria* growth. Specific compounds in the concentrate mix, such as readily fermentable carbohydrates, could promote the growth of certain bacterial groups. At the same time, grass with high fiber content may promote the growth of beneficial bacteria such as lactic acid bacteria and fiber decomposing bacteria, and inhibit the growth of some potential pathogenic bacteria such as *Proteobacteria* [41].

At the genus level, Liu et al [43] reported that *unclassified Ruminococcaceae UCG-005* and *Lachnospiraceae* were predominant in yak feces, while [44] identified *Ruminococcaceae UCG-005* and *uncultured Bacteroides* as the main bacteria. This aligns with our findings, where *Ruminococcaceae_UCG_005* and *uncultured_bacterium* were predominant. Differences from Liu et al [43] may relate to breed, age, and diet. *Ruminococcaceae_UCG_005* is involved in cellulose degradation and starch digestion, contributing to fiber breakdown and feed efficiency [45]. *Rikenellaceae_RC9_gut_group* promotes gut health by inhibiting harmful bacteria and supporting beneficial ones. *Alistipes*, which decompose cellulose and complex carbohydrates to generate short-chain FAs, were more abundant in the yaks in GS group, potentially explaining their higher FA levels [45]. *Family_XIII_UCG-001* is a bacterium associated with fiber degradation and short-chain FA production. Its abundance typically was increased with the amount of fiber, especially in relation to the fermentable components in pasture [46]. Therefore, in this study, the higher abundance of *Family_XIII_UCG-001* in the G group may be due to the animals primarily obtaining fiber from natural pasture, ensuring sufficient fiber for this bacterial group to utilize. *Erysipelatoclostridium* with other bacteria promotes the breakdown of complex carbohydrates, aiding nutrient absorption and maintaining intestinal integrity [47]. In this study, the GS group exhibited a higher abundance of *Erysipelatoclostridium* compared to the G group. This discrepancy could be attributed to the additional supplementation in the GS group. Furthermore, the higher N availability in the GS group might have supported the growth of nitrogen-utilizing bacteria, promoting a more efficient nutrient absorption process.

*Tuzzerella* was associated with nitrogen metabolism, particularly in promoting the utilization of proteins and AAs in the body. Its activity supports effective nutrient absorption and enhances metabolic health by improving fiber degradation and N utilization in ruminants [48,49]. In this study, *Tuzzerella* was found to be significantly more abundant in the GS group compared to the G group. This increased abundance was likely due to the supplementation of concentrate mix in the GS group, which provided additional fermentable carbohydrates and N. The availability of these nutrients likely supported the growth of *Tuzzerella* and other fiber-degrading bacteria, thereby enhancing fiber digestion. *Clostridium* ferments complex carbohydrates and fibers into VFA like acetate and propionate, key energy sources for cattle. Pinnell et al [50] reported a higher relative abundance of the genus *UCG-009* in the gut of cattle with high feed efficiency. Our results showed that the content of *UCG-009* bacteria in the yaks in GS group was significantly higher than that in the yaks in G group, which was consistent with the growth performance data.

In this study, no significant differences were observed in the fecal diversity indices. This might have been attributed to the stability of the gut microbial community, where short-term supplementary feeding did not significantly affect microbial richness or evenness [51]. Although supplementary feeding may have altered the structure of the microbial community, its impact on overall diversity was likely insufficient to induce detectable changes in these indices. Further research with longer durations or more intensive feeding protocols is needed to elucidate the long-term effects of supplementary feeding on gut microbiota diversity [52].

In this study, norank_f__*Oscillospiraceae* exhibited a significant negative correlation with Ca, P, and N, potentially due to its impact on intestinal pH. Gut microbiota ferment dietary substrates, producing short-chain FAs that lower luminal pH and affect mineral solubility. Excessive acidification can precipitate insoluble mineral complexes, reducing Ca and P bioavailability [53]. Additionally, norank_f__*Oscillospiraceae* may compromise intestinal epithelial integrity and immune responses. The gut barrier is essential for nutrient absorption, and certain bacterial taxa influence epithelial permeability and inflammatory signaling [54]. If norank_f__*Oscillospiraceae* induces low-grade inflammation or disrupts tight junctions, it may impair mineral transport across the epithelium. Kim et al [55] demonstrated that chronic inflammation downregulates key mineral transporters. These factors may explain the negative correlation observed in this study between norank_f__*Oscillospiraceae* and Ca, P, and N. Prevotellaceae_UCG-004 exhibited a significant positive correlation with butyric acid and isovaleric acid. Prevotellaceae are well known for their ability to degrade complex carbohydrates, particularly plant-derived fibers, converting them into fermentation end-products such as SCFAs. Butyrate, a major SCFA, serves as a primary energy source for colonic epithelial cells, enhances intestinal barrier integrity, and exhibits anti-inflammatory properties [56]. The observed positive correlation between Prevotellaceae_UCG-004 and butyrate suggests that this bacterium may contribute to butyrate biosynthesis, either directly or through cross-feeding interactions with other microbial species. Additionally, the positive correlation with isovaleric acid indicates that Prevotellaceae_UCG-004 may also be involved in protein fermentation. Isovaleric acid, a branched-chain SCFA, is derived from the microbial degradation of branched-chain AAs. This suggests that, in addition to fiber fermentation, Prevotellaceae_UCG-004 may also be involved in protein degradation or microbial biomass turnover.

Christensenellaceae_R-7_group exhibited a significant negative correlation with butyrate, suggesting that this bacterium may interfere with the activity of other microbes involved in fiber fermentation, particularly those producing butyrate, such as *Faecalibacterium prausnitzii* and *Roseburia* spp. [57]. If Christensenellaceae_R-7_group competes with or suppresses the growth of these butyrate-producing bacteria, it could lead to a reduction in butyrate production [58]. The observed negative correlation may also arise from metabolic cross-talk between Christensenellaceae_R-7_group and other microbial taxa [59]. Given the prevalence of cross-feeding in gut microbiota, Christensenellaceae_R-7_group may secrete metabolites that suppress the growth or activity of butyrate-producing microbes. Another potential explanation is that Christensenellaceae_R-7_group may influence the regulation of gut pH, a critical factor in SCFA production. Certain bacteria alter luminal pH through their metabolic activities, affecting the solubility and bioavailability of substrates for butyrate-producing bacteria. A lower pH may favor the growth of other microbial types while inhibiting butyrate producers. Candidatus_*Soleaferrea* exhibited a significant negative correlation with isobutyric acid, a branched-chain short-chain fatty acid (BCFA) typically produced during the microbial degradation of branched-chain AAs [60]. These findings suggest that Candidatus_*Soleaferrea* may modulate isobutyric acid levels by inhibiting key microbial producers or altering associated fermentation pathways. Candidatus_*Soleaferrea* may compete with other microbes involved in protein fermentation, thereby reducing isobutyric acid production. Many gut microbes, particularly those from the *Firmicutes* and *Bacteroidetes* phyla, contribute to protein fermentation and subsequent BCFA production. If Candidatus_*Soleaferrea* interacts with or suppresses the growth of these protein-fermenting bacteria, it could lead to a shift in the microbial fermentation pattern, resulting in a decrease in isobutyric acid levels. Additionally, Candidatus_*Soleaferrea* may influence the microbial community structure in a way that indirectly reduces isobutyric acid production. By promoting the growth of other bacterial groups that produce other SCFAs, Candidatus_*Soleaferrea* can alter the overall fermentation product profile in the gut, potentially displacing isobutyric acid-producing bacteria and reducing their activity.

## CONCLUSION

**Figure 4:**
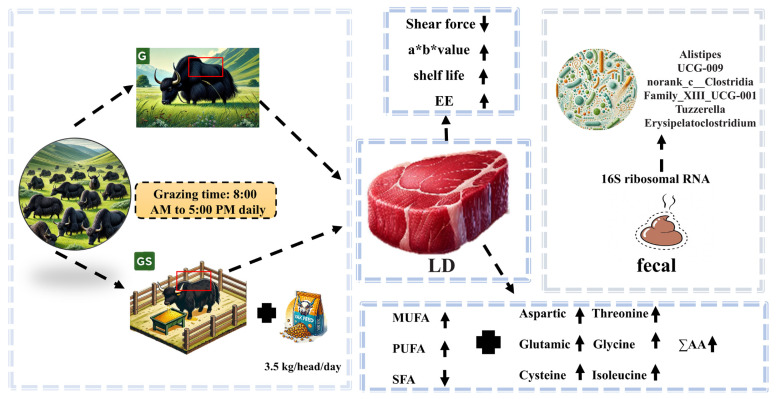
Diagram illustrating the impact of yaks in the G group and GS group on LD muscle quality and gut microbiota composition. Muscle quality indicators include shear force, a* (redness), b* (yellowness), and shelf life. Gut microbiota composition was analyzed at the genus level using 16S rRNA sequencing. Dashed arrows indicate processes or logical relationships, while solid arrows indicate the direction of change (↑: increased, ↓: decreased). G, grazing group; GS, grazing and supplementary feeding group; EE, ether extract; LD, longissimus dorsi; MUFA, monounsaturated fatty acids; PUFA, polyunsaturated fatty acids; SFA, saturated fatty acids; ΣAA, sum of amino acids.

The results and underlying mechanisms of these improvements are illustrated in Figure 4, highlighting key findings related to enhanced meat quality, including reductions in shear force, increased a* and b* values, fat content, and shelf life. Additionally, the figure summarizes the changes in gut microbiota composition.

This study demonstrates that post-grazing supplementation with a concentrate mix significantly improves the growth performance, meat quality, and nutrient utilization of yaks. Specifically, it enhances meat tenderness, color stability, and FA composition—especially by increasing MUFAs and PUFAs—thereby improving both sensory characteristics and nutritional value. Importantly, this is the first study to report that concentrate mix supplementation alters specific gut microbial taxa (e.g., *Tuzzerella*, *UCG-009*, *Alistipes*), which are associated with fiber degradation, nitrogen metabolism, and FA biosynthesis, providing insight into the microbial mechanisms underlying these improvements.

Moreover, the observed increase in fecal nitrogen content and its correlation with microbial composition suggested enhanced nitrogen retention and recycling potential in alpine grazing systems. These findings collectively contributed new evidence supporting the integration of concentrate supplementation into yak feeding strategies, not only for improving meat quality, but also for optimizing nutrient cycling and promoting sustainable yak husbandry in high-altitude regions.

## Figures and Tables

**Table 1:** Effect of different grazing practice on growth performance of yaks

Items	Group		Cohen’s *d*	p-value (group)	p-value (group×time)

	G	GS			
Initial body weight (kg)	218.31±1.02	216.23±1.02	0.589	0.182	
Interval body weight (kg)	234.75±1.02	244.44±1.02	2.742	<0.001	<0.001
Final body weight (kg)	249.94±1.02	271.85±1.02	6.201	<0.001	<0.001
Average daily gain (kg/ day)	0.46±0.023	0.86±0.023	7.939	<0.001	

Data are presented as mean±SEM.

Cohen’s *d* is reported to show the effect size between groups.

p-values represent the significance level for group×time interaction effects based on linear mixed models.

G, grazing group; GS, grazing and supplementary feeding group; SEM, standard error of the mean.

**Table 2:** Effects of different grazing practices on meat quality and nutritional composition of yak longissimus dorsi muscle

Items	Group		SEM	Cohen’s *d*	p-value

	G	GS			
pH _45 min_	6.37	6.39	0.053	0.109	0.772
pH _24 h_	5.78	5.79	0.031	0.093	0.727
L* (lightness)	30.63	30.68	0.548	0.026	0.920
a* (redness)	11.78	13.88	0.640	0.947	0.008
b* (yellowness)	8.84	9.74	0.379	0.686	0.039
Cooking loss (%)	31.33	28.56	0.944	0.847	0.100
Drip loss (%)	3.63	3.56	0.562	0.036	0.903
Shear force (N)	9.62	9.11	0.221	0.666	0.047
Moisture (%)	71.94	72.70	0.786	0.279	0.372
EE (%)	1.41	1.73	0.117	0.790	0.030
CP (%)	25.11	25.05	0.476	0.036	0.909
Ash (%)	1.90	1.75	0.117	0.370	0.253

Cohen’s *d* is reported to show the effect size between groups.

G, grazing group; GS, grazing and supplementary feeding group; SEM, standard error of the mean; EE, ether extract; CP, crude protein.

**Table 3:** Effect of different grazing practice on meat AA content of yaks

Items	Group		SEM	Cohen’s *d*	p-value

	G	GS			
Aspartic	2.06	2.15	0.044	0.590	0.064
Threonine	1.05	1.11	0.025	0.693	0.048
Serine	0.82	0.83	0.004	0.722	0.131
Glutamic	3.30	3.47	0.043	1.141	<0.001
Proline	0.28	0.28	0.004	0.000	0.976
Glycine	0.91	0.93	0.004	1.443	<0.001
Alanine	1.30	1.33	0.180	0.048	0.176
Cysteine	0.13	0.14	0.026	0.111	0.005
Valine	1.18	1.16	0.038	0.152	0.640
Methionine	0.61	0.62	0.006	0.481	0.171
Isoleucine	1.04	1.10	0.014	1.237	<0.001
Leucine	1.96	2.00	0.030	0.385	0.204
Tyrosine	0.64	0.64	0.003	0.000	0.819
Phenylalanine	0.94	0.94	0.007	0.000	0.737
Lysine	2.10	2.20	0.057	0.506	0.120
Histidine	0.88	0.87	0.007	0.412	0.465
Arginine	1.30	1.30	0.016	0.000	0.935
∑AA	20.53	21.09	0.167	0.968	<0.001

Cohen’s *d* is reported to show the effect size between groups.

AA, amino acid; G, grazing group; GS, grazing and supplementary feeding group; SEM, standard error of the mean; ∑AA, sum of amino acids.

**Table 4:** Effect of different grazing practice on meat fatty acid content of yaks

Items	Group		SEM	Cohen’s *d*	p-value

	G	GS			
C6:0	2.12	2.00	0.008	4.330	<0.001
C8:0	0.8	0.78	0.009	0.642	0.032
C10:0	2.08	2.08	0.048	0.000	0.974
C12:0	0.35	0.31	0.006	1.925	<0.001
C13:0	0.23	0.20	0.004	2.165	<0.001
C14:0	0.72	0.69	0.007	1.237	<0.001
C15:0	4.16	4.06	0.031	0.931	<0.001
C16:0	26.69	27.03	0.066	1.487	<0.001
C17:0	3.16	3.34	0.047	1.528	<0.001
C18:0	12.93	12.29	0.933	0.198	<0.001
C19:0	0.64	0.60	0.007	1.650	<0.001
C20:0	2.56	6.03	0.026	38.527	<0.001
C22:0	0.60	0.61	0.054	0.053	0.316
C24:0	0.56	0.53	0.006	1.443	<0.001
C26:0	0.14	0.15	0.003	0.962	<0.001
ΣSFA	57.75	55.27	0.110	6.508	<0.001
C10:1	0.30	0.32	0.007	0.825	<0.001
C14:1	0.49	0.51	0.006	0.962	<0.001
C15:1	0.11	0.13	0.003	1.925	<0.001
C16:1	6.28	6.50	0.628	0.101	0.317
C17:1	0.46	0.48	0.005	1.155	<0.001
C18:1	26.49	28.38	0.118	4.624	<0.001
C19:1	1.47	1.51	0.008	1.443	<0.001
C20:1	1.30	1.36	0.136	0.127	<0.001
C22:1	0.32	0.33	0.004	0.722	<0.001
C24:1	0.24	0.24	0.004	0.000	0.763
∑MUFA	37.47	39.77	0.121	5.487	<0.001
C18:2	2.17	2.27	0.040	0.722	0.020
C18:3	0.70	0.71	0.006	0.481	0.111
C20:3	0.12	0.13	0.003	0.962	0.084
C20:4	0.16	0.17	0.002	1.443	<0.001
C20:5	0.49	0.47	0.024	0.241	0.535
C22:6	1.14	1.20	0.201	0.086	<0.001
∑PUFA	4.78	4.96	0.045	0.086	<0.001

Cohen’s *d* is reported to show the effect size between groups.

G, grazing group; GS, grazing and supplementary feeding group; SEM, standard error of the mean; ∑SFA, sum of saturated fatty acid; ∑MUFA, sum of monounsaturated fatty acid; ∑PUFA, sum of polyunsaturated fatty acid.

**Table 5:** Effect of different grazing practices on volatile fatty acid composition and constant elements in yak feces

Items	Group		SEM	Cohen’s *d*	p-value

	G	GS			
Ca (%)	1.29	1.59	0.100	0.867	0.013
P (%)	0.37	0.38	0.012	0.083	0.446
N (%)	2.15	2.31	0.090	0.178	0.045
VFA (mM)					
Acetic acid	53.16	60.81	5.067	0.151	0.157
Propionic acid	11.03	13.62	1.900	0.136	0.196
Isobutyric acid	0.39	0.39	0.115	0.000	0.993
Butyric acid	1.63	2.34	0.548	0.129	0.217
Isovaleric acid	0.40	0.44	0.117	0.034	0.722
Valeric acid	0.43	0.42	0.054	0.019	0.890
A:P	4.99	4.62	0.503	0.074	0.473
Total VFA	66.72	78.10	7.124	0.160	0.136

Cohen’s *d* is reported to show the effect size between groups.

G, grazing group; GS, grazing and supplementary feeding group; SEM, standard error of the mean; VFA, volatile fatty acids; A:P, acetic acid to propionic acid ratio.

**Table 6:** Effect of different grazing practice on alpha diversity in yak feces

Items	Group		SEM	Cohen’s *d*	p-value

	G	GS			
Ace	1,117.25	1,106.91	57.713	0.057	0.860
Chao	1,105	1,094	55.564	0.057	0.848
Shannon	6.21	6.20	0.069	0.014	0.962
Simoson	0.004	0.004	0.001	0.000	0.677
Sobs	1,096	1,085	52.393	0.057	0.826

Cohen’s *d* is reported to show the effect size between groups.

G, grazing group; GS, grazing and supplementary feeding group; SEM, standard error of the mean.
